# Comparison of treatment outcome between first‐line combination immunotherapy (anti‐PD‐L1 or anti‐PD1) with or without chemotherapy and chemotherapy alone in advanced non‐small cell lung cancer patients in tertiary care hospital

**DOI:** 10.1002/cam4.70007

**Published:** 2024-07-18

**Authors:** Danainut Naratornsirakul, Busyamas Chewaskulyong, Sarawut Kongkarnka, Songporn Oranratnachai

**Affiliations:** ^1^ Division of Medical Oncology, Department of Internal Medicine, Faculty of Medicine Chiang Mai University Chiang Mai Thailand; ^2^ Department of Pathology, Faculty of Medicine Chiang Mai University Chiang Mai Thailand; ^3^ Sriphat Medical Center, Faculty of Medicine Chiang Mai University Chiang Mai Thailand

**Keywords:** anti‐Pd1 immunotherapy, anti‐PD‐L1, immunotherapy, non‐small cell lung cancer

## Abstract

**Background:**

Despite promising outcomes of first‐line immunotherapy with or without chemotherapy in advanced non‐small cell lung cancer (NSCLC), limited accessibility due to reimbursement was remain the problem in low to middle income countries. This study aimed to evaluate real‐world effectiveness of immunotherapy in patients with advanced NSCLC in Northern Thailand.

**Method:**

A retrospective, single‐centered cohort, was conducted. Patients with advanced NSCLC who underwent PD‐L1 testing (excluding EGFR and ALK mutations) and were treated with immunotherapy or without chemotherapy or chemotherapy alone were included. The primary end point was progression‐free survival (PFS). The secondary endpoints were overall survival (OS), objective response rate (ORR), and adverse events.

**Results:**

A total of 123 patients, of which 21 patients received immunotherapy‐based regimen and 102 patients received chemotherapy alone. The median PFS was 11.9 months in immunotherapy‐based group compared to 5.93 months in the chemotherapy group, with a. hazard ratio (HR) of 0.4 (95% confidence interval [CI], 0.23 to 0.68; *p* = 0.001). Similarly, the median OS was 26.68 months in the immunotherapy‐based group and 11.21 months in the chemotherapy group, with HR of 0.42 (95% CI 0.22–0.8; *p* = 0.009). ORRs were significantly higher in the immunotherapy‐based group, with 65% of patients showing a response compared to 32% in the chemotherapy group (*p* = 0.006).

**Conclusion:**

The result of this real‐world study in patients with advanced stage NSCLC indicate that first‐line immunotherapy‐based regimen was associated with significantly greater PFS, OS, and ORR with a safety profile consistent with pivotal studies.

## 1 INTRODUCTION

Lung cancer is one of the most diagnosed cancers, and it is the most leading cause of death worldwide.^1^ Newly diagnosed lung cancer occurs with approximately 2.2 million patients per year, constituting 11.4% and resulting in 1.8 million deaths per year, representing 18% of all cancer‐related deaths. In Thailand, lung cancer is the second most diagnosed cancer accounting for 20,395‐cases (16.3%).^1^, ^2^

Nowadays, first‐line immunotherapy with or without chemotherapy is the standard treatment in non‐small cell lung cancer (NSCLC) who had no targeted oncogene driven therapy which show better efficacy and survival when compared to chemotherapy alone.^3^, ^4^, ^5^, ^6^, ^7^, ^8^, ^9^, ^10^, ^11^, ^12^, ^13^, ^14^, ^15^

The prevalence of programmed death‐ligand 1 (PD‐L1) positive NSCLC varies across races. Caucasian patients, 63% have PD‐L1 positive tumors, 33% of Chinese patients exhibit PD‐L1 positivity, smoking is a share risk factor that increases likelihood of PD‐L1 high expression.^16^, ^17^

Hence, researchers recognize the significance of real‐world data^18^, ^19^, ^20^, ^21^, ^22^, ^23^, ^24^ in elucidating the efficacy of immunotherapy. In Asian countries such as China, Japan, and Korea, there are real‐world data^25^, ^26^, ^27^, ^28^, ^29^ comprising 1390 treated patients with immunotherapy, either with or without chemotherapy demonstrate survival benefits consistent with clinical studies. Clinical studies typically enroll patients with favorable performance status, absence of or stable central nervous system (CNS) metastasis, and satisfactory organ function. Consequently, patients encountered in clinical practice may be excluded or inadequately represented in clinical trials. Therefore, our study aims to offer insights into the real‐world effectiveness and safety of immunotherapy utilization in upper middle income country.^30^

## 2 MATERIALS AND METHODS

### 2.1 Study design

A retrospective observational cohort study was conducted at Maharaj Nakorn Chiang Mai Hospital in Chiang Mai, Thailand which was tertiary care hospital in an upper middle‐income country. The study received approval from the Research Administration Section of Faculty of Medicine, Chiang Mai, University, Chiang Mai, Thailand (MED‐2566‐0338).

The electronic medical records were retrospectively reviewed. Patients who were at least 18‐years‐old with histologically or cytologically confirmed advanced‐stage NSCLC who underwent PD‐L1 testing and received systemic treatment at Maharaj Nakorn Chiang Mai hospital between January 1st, 2013 and December 31st, 2022 were included. Patients with epidermal growth factor receptor (EGFR) mutation and Anaplastic lymphoma kinase (ALK) translocation were excluded. Additionally, patients with history of others malignancy within 5 years and loss to follow‐up within 1 month were also excluded.

### 2.2 Objective

The primary objective was to compare progression‐free survival (PFS) between immunotherapy (Anti‐PD‐L1 or Anti‐PD1) with or without chemotherapy (Immunotherapy‐based group) and conventional chemotherapy (Chemotherapy group) in the first‐line treatment of advanced NSCLC.

Key secondary objectives were overall survival (OS), objective response rate (ORR), and adverse events (AEs) between immunotherapy‐based to chemotherapy group in the first‐line treatment of advanced NSCLC.

PFS was defined as the time from the start of systemic treatment until the date of disease progression or death, whichever came first. OS was defined as the time from the start of first‐line systemic treatment until death from any cause. An ORR was defined as a complete or partial response according to the RECIST version 1.1 definition.^31^ AEs was graded according to Common Terminology Criteria for Adverse Events (CTCAE) version 4.0.

### 2.3 Statistical analysis

Based on primary objective of PFS, we estimated that a sample of at least 165 patients with 66 events of disease progression or death would provide 80% power to detect a hazard ratio of 0.5 at a two‐sided alpha level of 0.05.

Baseline characteristics were descriptively reported, comparing between immunotherapy‐based group chemotherapy group were done using chi squared or fisher's exact test for categorical data and student *t*‐test for continuous data as appropriated. OS and PFS were estimated by Kaplan–Meier method. Univariate and multivariate Cox regression analysis was applied to identified variables associated with PFS and OS.

For categorical outcomes such as ORR and AEs, frequency and proportion were reported. Group comparisons of ORR were carried out using the Chi squared or Fisher's Exact test as appropriated.

## 3 RESULTS

### 3.1 Patients and treatment

From January 2013 to December 2023, in Maharaj Nakorn Chiang Mai hospital, newly diagnosed advanced lung cancer patients were 6834 patients. Of which 247 patients had PD‐L1 testing and underwent medical record review. A total of 123 patients received at least one dose of treatment, categorized into either the immunotherapy‐based group (21 patients) and the chemotherapy group (102 patients). A further 114 patients were excluded from the study due to not receiving treatment at Maharaj Nakorn Chiang Mai hospital. Additionally, 10 patients were excluded from the study because they were enrolled in a clinical trial.

The demographic characteristics of the patients were generally well balanced between the two groups, except for the PD‐L1 tumor proportional score (TPS) levels. Notably, PD‐L1 TPS ≥50% was more prevalent in the immunotherapy‐based group (76.19% vs. 26.47%), whereas PD‐L1 TPS 1%–49% and PD‐L1 TPS <1% were more common in the chemotherapy group (0% vs. 19.61% and 23.81% vs. 53.92%, respectively), with a *p*‐value <0.001. Differences in first‐line chemotherapy regimen between the two groups were observed as presented in Table 1. Platinum/pemetrexed regimen was commonly used in the immunotherapy‐based group, whereas platinum with taxane‐based regimen was commonly used chemotherapy group (Table 1).

**TABLE 1:** Baseline characteristics of patients.

Characteristic, *N* (%)	Immunotherapy with or without chemotherapy (*N* = 21)	Chemotherapy (*N* = 102)	*p*‐value
Age, median (range), years	64.6 (27.6–88.85)	65.4 (28.5–86.1)	0.814
Gender			
Male	16 (76.19%)	66 (64.71%)	0.309
Female	5 (23.81%)	36 (35.29%)	
Reimbursement			
Universal coverage	3 (14.29%)	37 (36.27%)	0.124
Social security scheme	1 (4.76%)	6 (5.88%)	
CSMBS	17 (80.95%)	59 (57.84%)	
ECOG			
0	5 (23.81%)	15 (14.71%)	0.199
1	13 (61.9%)	81 (79.41%)	
2	3 (14.29%)	5 (4.9%)	
3	0 (0%)	1 (0.98%)	
Smoking status			
Never smoker	9 (42.86%)	32 (31.37%)	0.305
Former smoker	11 (52.38%)	53 (51.96%)	
Current smoker	1 (4.76%)	17 (16.67%)	
Metastasis site			
Brain	5 (23.81%)	20 (19.61%)	0.130
Liver	4 (19.05%)	18 (17.65%)	0.545
Bone	9 (42.86%)	34 (33.33%)	0.405
Adrenal gland	2 (9.52%)	23 (22.55%)	0.145
Lung	14 (66.67%)	64 (62.75%)	0.734
Pleura	8 (38.1%)	36 (35.29%)	0.807
Other	13 (61.9%)	43 (41.16%)	0.098
Histology			
Adenocarcinoma	15 (71.43%)	77 (75.49%)	0.713
Squamous cell carcinoma	6 (28.57%)	23 (22.55%)	
Others	0 (0%)	2 (1.96%)	
PD‐L1 clone			
SP142	2 (9.52%)	12 (11.76%)	0.559
SP263	1 (4.76%)	4 (3.92%)	0.615
22C3	18 (85.71%)	91 (89.22%)	0.441
PD‐L1 TPS			
< 1%	5 (23.81%)	55 (53.92%)	<0.001
1%–49%	0 (0%)	20 (19.61%)	
≥50%	16 (76.19%)	27 (26.47%)	
BMI (kg/m^2^)			
<18.5	4 (19.05%)	23 (22.55%)	0.893
18.5–24.9	13 (61.9%)	65 (63.73%)	
25–29.9	3 (14.29%)	10 (9.8%)	
>30	1 (4.76%)	4 (3.92%)	
Previous therapy			
Surgery	3 (14.29%)	13 (12.75%)	0.541
Adjuvant/neoadjuvant chemotherapy	1 (4.76%)	5 (4.9%)	0.728
Radiotherapy	3 (14.29%)	6 (5.88%)	0.181
CCRT	1 (4.76%)	3 (2.94%)	0.532
First line chemotherapy in advanced/metastasis			
Platinum/(Nab‐) Paclitaxel	5 (23.81%)	59 (57.84%)	<0.001
Platinum/pemetrexed	7 (33.33%)	24 (23.53%)	
Platinum/gemcitabine	0 (0%)	7 (6.86%)	
Other	1 (4.76%)	12 (11.76%)	
No chemotherapy	8 (38.1%)	0 (0%)	
Immunotherapy			
Atezolizumab	1 (4.76%)	0 (0%)	<0.001
Pembrolizumab	19 (90.48%)	0 (0%)	
Nivolumab	1 (4.76%)	0 (0%)	

Abbreviations: BMI, body mass index; CCRT, concurrent chemoradiation; CSMBS, Civil Servant Medical Benefit Scheme; ECOG, the Eastern Cooperative Oncology Group; PD‐L1, programmed death‐ligand 1; TPS, tumor proportional score.

**FIGURE 1:**
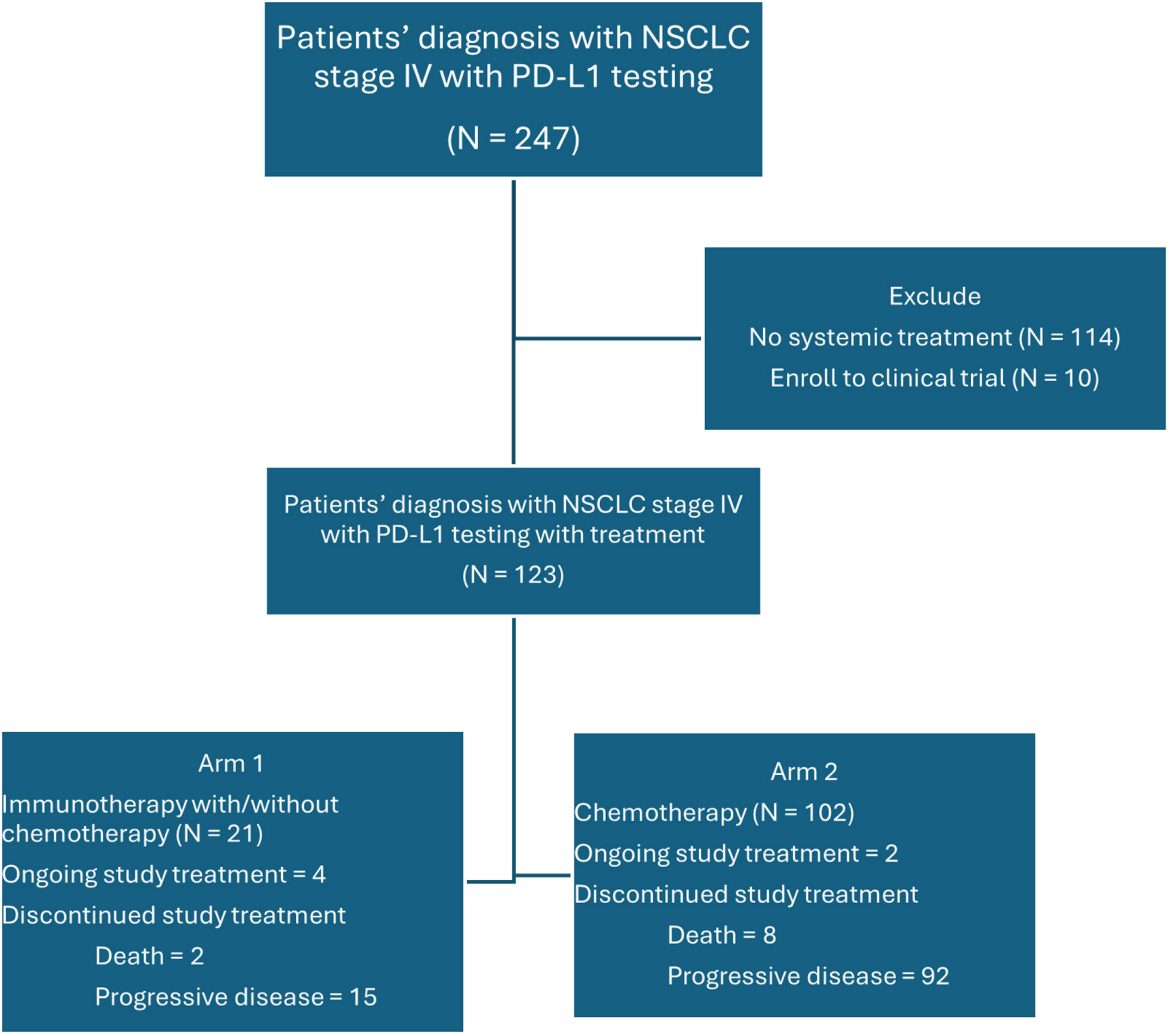
Trial profile.

At the time of data cutoff, four patients (19%) in the immunotherapy‐based group and two patients (1.9%) in the chemotherapy group were still receiving study treatment. Seventeen (81%) patients in the immunotherapy‐based group and 100 patients (98.1%) in the chemotherapy group had discontinued the study treatment, with the most common reason being disease progression. The details of trial profile are shown in Figure 1.

### 3.2 Efficacy

**FIGURE 2:**
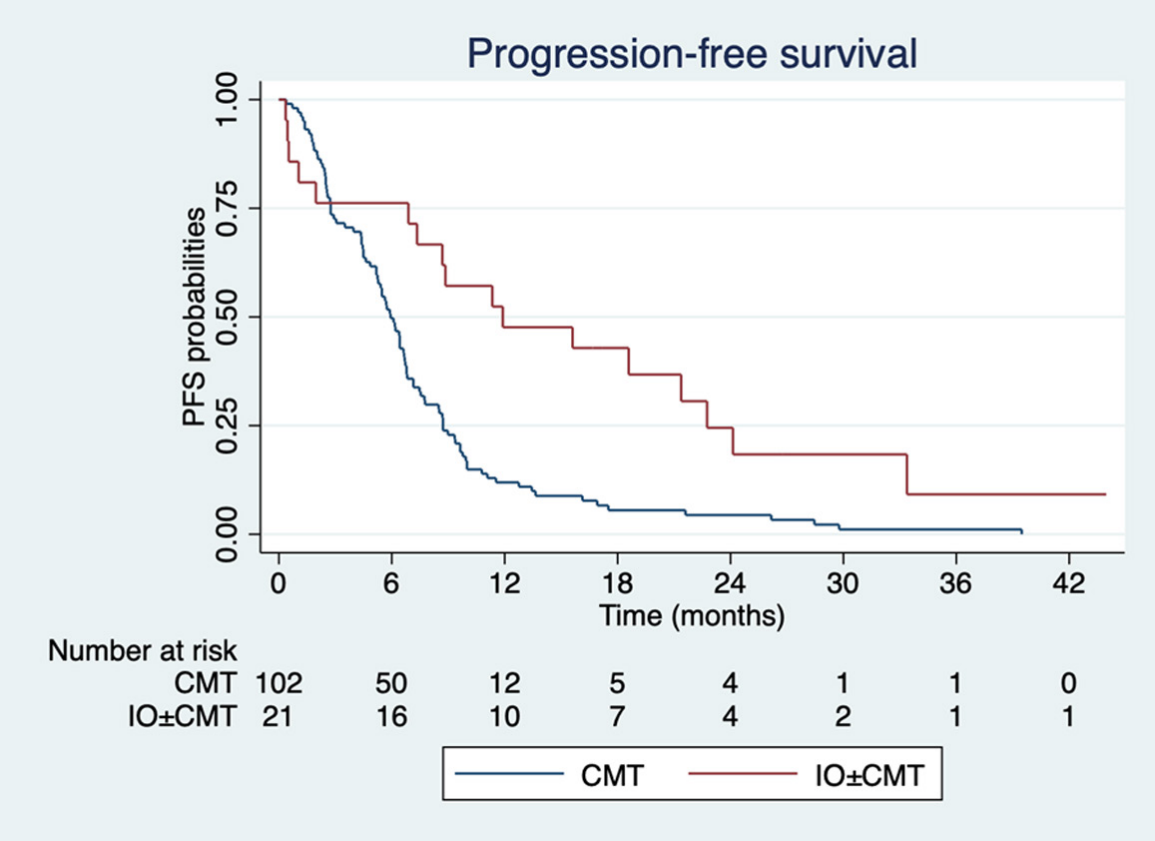
Kaplan–Meier estimates PFS between immunotherapy‐based group (red line) and chemotherapy group (blue line).

The median PFS was 11.9 months in the immunotherapy‐based group and 5.9 months in the chemotherapy group (Figure 2). The hazard ratio (HR) for disease progression or death was 0.4 (95% confidence interval [95% CI], 0.23 to 0.68; *p* = 0.001), indicating a significant improvement in PFS with the immunotherapy‐based regimen.

Univariate cox‐regression analysis across various factors was conducted. A significant factor associated with PFS was present of adrenal metastasis, the unadjusted PFS HR was 2.00 (95% CI 1.32–3.31; *p* = 0.001), indicating a significant worsening of PFS in patients with adrenal metastasis. Further PFS estimates for other co‐variables were presented in Table 2.

**TABLE 2:** Univariate and multivariate cox‐regression analysis of PFS.

Variables	*N*	mPFS (mo)	Univariate analysis		Multivariate analysis	
			HR (95% CI)	*p*‐value	HR (95% CI)	*p*‐value
Treatment						
Immunotherapy	21	11.9	0.40 (0.23–0.68)	0.001	0.45 (0.25–0.81)	0.008
Chemotherapy	102	5.93	Ref		Ref	
Gender						
Male	82	6.42	0.83 (0.56–1.22)	0.364		
Female	41	6.39	Ref			
ECOG‐PS						
0–1	114	6.42	Ref			
2–4	9	3.04	1.28 (0.64–2.54)	0.474		
Smoking						
Current/ex‐smoker	82	6.19	0.79 (0.53–1.18)	0.256		
Never smoker	41	6.62	Ref			
Histology						
Adenocarcinoma	92	6.78	Ref		Ref	
Squamous cell carcinoma	29	5.47	1.34 (0.87–2.06)	0.178	1.35 (0.86–2.11)	0.183
Other	2	1.37	3.29 (0.79–13.6)	0.099	3.5 (0.78–16.16)	0.099
Metastasis^a^						
Lung	78	6.62	0.82 (0.56–1.2)	0.330		
Liver	22	5.47	1.53 (0.95–2.45)	0.076	1.27 (0.75–2.13)	0.362
Bone	43	5.7	1.1 (0.75–1.61)	0.613		
Brain	25	6.42	1.1 (0.67–1.81)	0.691		
Adrenal	25	4.85	2.00 (1.32–3.31)	0.001	1.88 (1.14–3.09)	0.013
Other	56	6.19	1.08 (0.75–1.56)	0.646		
PD‐L1 TPS						
<1%	60	5.93	Ref			
1%–49%	20	6.78	0.64 (0.37–1.1)	0.113	0.66 (0.37–1.16)	0.15
≥50%	43	7.47	0.51 (0.33–0.79)	0.003	0.62 (0.39–1.00)	0.053
BMI (kg/m^2^)						
<18.5	27	4.39	Ref			
18.5–24.9	78	6.39	0.74 (0.47–1.16)	0.195		
25–29.9	13	7.73	0.45 (0.23–0.90)	0.025		
>30	5	7.77	0.74 (0.28–1.96)	0.557		
First‐line regimen						
Combination IO	13	11.9	0.36 (0.19–0.71)	0.003		
Single IO	8	7.3	0.35 (0.16–0.80)	0.013		
Platinum‐doublet	90	5.7	Ref			
Single CMT	8	6.78	0.71 (0.34–1.47)	0.360		
Other CMT	4	6.65	0.45 (0.16–1.25)	0.130		

Abbreviations: BMI, body mass index; CMT, chemotherapy; ECOG‐PS, the Eastern Cooperative Oncology Group‐Performance status; IO, Immunotherapy; PD‐L1, programmed death‐ligand 1; TPS, Tumor proportional score.

^a^ HR in the table was HR of organ metastasis compared to no metastasis.

The co‐variables with *p*‐value <0.10 from univariate cox‐regression analysis were considered in multivariate analysis. Thus, treatment group, histology types, liver metastasis, adrenal metastasis, and PD‐L1 expression levels were included in the model. After adjusted in multivariate analysis, PFS was significant longer in immunotherapy‐based group compared to chemotherapy group with adjusted PFS HR of 0.45 (95% CI 0.25–0.81; *p* = 0.008).The adjusted PFS HR for histology type was 1.35 (95% CI 0.86–2.11) for squamous cell carcinoma and 3.5 (95% CI 0.78–16.16) for other histology types. For liver and adrenal metastasis, the adjusted PFS HR was 1.27 (95% CI 0.75–2.13) and 1.88 (95% CI 1.14–3.09), respectively, indicating a significant worsening of PFS in patients with adrenal metastasis. Among PD‐L1 expression levels, comparing to PD‐L1 TPS <1%, the adjusted PFS HR was 0.66 (95% CI 0.37–1.16) for PD‐L1 TPS 1–49% and 0.62 (95% CI 0.39–1.00) for PD‐L1 TPS ≥50%.

**FIGURE 3:**
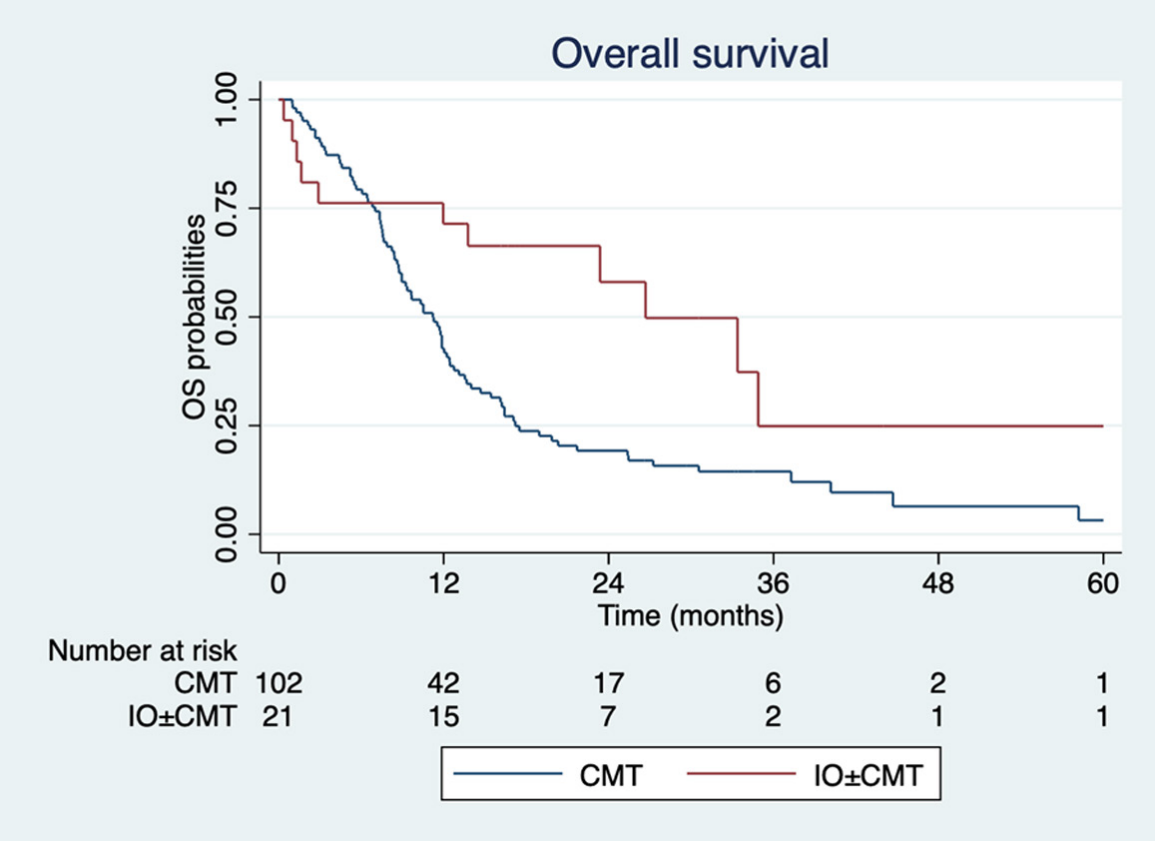
Kaplan–Meier estimates OS between immunotherapy‐based group (red line) and chemotherapy group (blue line).

The median OS was 26.6 months in the immunotherapy‐based group and 11.2 months in the chemotherapy group (Figure 3). The HR for death was 0.42 (95% CI 0.22–0.80; *p* = 0.009), indicating a significant improvement in OS with the immunotherapy‐based regimen. This HR corresponds to a 58% reduction in the risk of death from any cause.

Univariate cox‐regression analysis across various factors was conducted. (Table 3) A significant variables associated with OS were histologic subtype, BMI group, present of metastasis to lung, liver, brain, and adrenal gland. The co‐variables with *p*‐value <0.1 from univariate analysis were adjusted in multivariate analysis except metastatic site. The metastatic sites were not included because of multicollinearity. We also included co‐variable of any line of immunotherapy in to multivariate model to explore the effect adding immunotherapy to first‐line treatment compare with later‐line of treatment.

**TABLE 3:** Univariate and multivariate cox‐regression analysis of OS.

Variables	*N*	mOS (mo)	Univariate analysis		Multivariate analysis	
			HR (95% CI)	*p*‐value	HR (95% CI)	*p*‐value
Treatment						
IO	21	26.68	0.42 (0.22–0.8)	0.009	0.64 (0.29–1.38)	0.261
CMT	102	11.21	Ref		Ref	
Gender						
Male	82	12.22	0.92 (0.61–1.41)	0.733		
Female	41	11.21	Ref			
ECOG‐PS						
0–1	114	11.96	Ref		Ref	
2–4	9	8.72	2.01 (0.97–4.18)	0.060	1.57 (0.7–3.49)	0.268
Smoking						
Current/ex‐smoker	82	11.86	1.02 (0.66–1.56)	0.911		
Never smoker	41	11.96	Ref			
Histology						
Adenocarcinoma	92	12.42	Ref		Ref	
Squamous cell carcinoma	29	9.63	1.38 (0.86–2.2)	0.171	1.48 (0.9–2.42)	0.118
Other	2	2.65	4.83 (1.15–20.26)	0.031	4.64 (1.03–20.7)	0.045
Metastasis^a^						
Lung	78	13.11	0.57 (0.38–0.86)	0.008		
Liver	22	8.72	1.71 (1.05–2.79)	0.031		
Bone	43	11.54	1.47 (0.97–2.22)	0.063		
Brain	25	11.86	1.80 (1.06–3.05)	0.029		
Adrenal	25	7.47	2.34 (1.46–3.75)	<0.001		
Other	56	11.86	1.09 (0.73–1.63)	0.641		
PD‐L1 TPS						
<1%	60	10.52	Ref		Ref	
1%–49%	20	13.11	0.59 (0.32–1.11)	0.104	0.69 (0.36–1.32)	0.267
≥50%	43	12.75	0.65 (0.41–1.02)	0.066	0.69 (0.41–1.14)	0.155
BMI (kg/m^2^)						
<18.5	27	7.4	Ref		Ref	
18.5–24.9	78	11.96	0.51 (0.31–0.83)	0.007	0.52 (0.3–0.88)	0.017
25–29.9	13	25.44	0.23 (0.1–0.53)	0.001	0.26 (0.11–0.61)	0.002
>30	5	13.54	0.55 (0.2–1.49)	0.245	0.58 (0.21–1.64)	0.312
First‐line regimen						
Combination IO	13	NR	0.29 (0.11–0.72)	0.008		
Single IO	8	23.37	0.62 (0.27–1.44)	0.274		
Platinum‐doublet	90	10.52	Ref			
Single CMT	8	11.21	0.70 (0.28–1.74)	0.450		
Other CMT	4	11.77	0.70 (0.25–1.93)	0.500		
Any line immunotherapy						
Yes	55	17.21	0.47 (0.31–0.71)	<0.001	0.62 (0.39–0.99)	0.047
No	68	8.95	Ref		Ref	

Abbreviations: BMI, body mass index; CMT, chemotherapy; ECOG‐PS, the Eastern Cooperative Oncology Group‐Performance status; IO, immunotherapy; PD‐L1, programmed death‐ligand 1; TPS, tumor proportional score.

^a^ HR in the table was HR of organ metastasis compared to no metastasis.

Across ECOG‐PS, the unadjusted OS HR was 2.01 (95% CI 0.97–4.18; *p* = 0.06) for ECOG‐PS 2–4 compared to patients ECOG 0–1. For smoking status, the unadjusted OS HR was 1.02 (95% CI 0.66–1.56; *p* = 0.911) in compared to non‐smoking patients.

Patients with other histology types exhibited significantly poorer OS compared to adenocarcinoma, with median OS of 2.65 months versus 12.42 months (HR for death 4.83 [95% CI 1.15–20.26; *p* = 0.031]).

Regarding metastatic sites, the unadjusted OS HR of adrenal metastasis compared to no adrenal metastasis was 2.34 (95% CI 1.46–3.75; *p* < 0.001), indicating a significant worsening of OS in adrenal metastasis patients. Same as patients with liver and brain metastasis that have significant worsening of OS with HRs for death of 1.71 (95% CI 1.05–2.79; *p* = 0.031) and 1.8 (95% CI 1.06–3.05; *p* = 0.029), respectively.

Across PD‐L1 expression levels, the unadjusted HR for death was 0.59 (95% CI 0.32–1.11; *p* = 0.104) in patients with PD‐L1 expression levels 1%–49% and 0.65 (95% CI 0.41–1.02; *p* = 0.066) in patients with PD‐L1 expression levels ≥50%, compared to patients with patients with PD‐L1 expression levels less than 1%.

Regarding BMI status, patients with a BMI of 18.5–24.9 kg/m^2^ nd 25–29.9 kg/m^2^ appeared to have better OS compared to those with a BMI less than 18.5 kg/m^2^ with HR for death of 0.51 (95% CI 0.31–0.83; *p* = 0.007) and 0.23 (95% CI 0.1–0.53; *p* = 0.001), respectively. However, this effect was not observed in patients with BMI greater than 30 kg/m^2^with HR for death of 0.55 (95% CI 0.2–1.49; *p* = 0.245).

After adjusted in multivariate analysis, first‐line treatment with immunotherapy‐based regimen was not significant factor associated with OS, the adjusted HR was 0.64 (95% CI 0.29–1.38; *p* = 0.261) for immunotherapy‐based group compared to chemotherapy alone. Whereas, patients who are eligible for immunotherapy during their treatment (any line of immunotherapy) have notably longer OS compared to those who are not eligible. The adjusted HR for death were 0.62 (95% CI 0.39–0.99; *p* = 0.047) (Table 3).

An ORR was observed in 65% of patients in the immunotherapy‐based group and in 32% of patients in the chemotherapy group (*p* = 0.006). Notably, ORR varied across subgroups, with a notably higher magnitude of immunotherapy benefit observed in PD‐L1 high compared to PD‐L1 negative subgroups (73.33% and 40%, respectively).

The clinical benefit rate (CBR) was higher in the immunotherapy‐based group compared to the chemotherapy group, although it did not reach significance. Table 4.

**TABLE 4:** Summary of response.

Best response	All patients (*N* = 123)		TPS <1% (*N* = 60)		TPS 1%–49% (*N* = 20)		TPS ≥ 50% (*N* = 43)	
	IO (*N* = 21)	CMT (*N* = 102)	IO (*N* = 5)	CMT (*N* = 55)	IO (*N* = 0)	CMT (*N* = 20)	IO (*N* = 16)	CMT (*N* = 27)
CR	1 (4.76%)	1 (0.98%)	0 (0%)	0 (0%)	0 (0%)	0 (0%)	1 (6.25%)	1 (3.7%)
PR	12 (57.14%)	31 (30.39%)	2 (40%)	20 (36.36%)	0 (0%)	6 (30%)	10 (62.5%)	5 (18.52%)
SD	3 (14.29%)	38 (37.25%)	1 (20%)	19 (34.55%)	0 (0%)	10 (50%)	2 (12.5%)	9 (33.33%)
PD	4 (19.05%)	29 (28.43%)	2 (40%)	14 (25.45%)	0 (0%)	3 (15%)	2 (12.5%)	12 (44.44%)
NE	1 (4.76%)	3 (2.94%)	0 (0%)	2 (3.64%)	0 (0%)	1 (5%)	1 (6.25%)	0 (0%)
ORR	13 (65%)	32 (32.32%)	2 (40%)	20 (37.74%)	0 (0%)	6 (31.58%)	11 (73.33%)	6 (22.22%)
*p*‐value	0.006		0.635		—		0.002	
CBR	16 (80%)	70 (70.7%)	3 (60%)	39 (73.58%)	0 (0%)	16 (84.21%)	13 (86.66%)	15 (55.55%)
*p*‐value	0.29		0.424		—		0.041	

*Note*: Data are *n* (%).

Abbreviations: CBR, clinical benefit rate; CR, complete response; NE, not evaluated; PD, progression of disease; PR, partial response; SD, stable of disease.

Among patients in the immunotherapy‐based group, 9 out of 21 (42.8%) received subsequent second‐line anticancer therapies, contrasting with 59 out of 102 (57.8%) in the chemotherapy group. Notably, patients in the immunotherapy‐based group predominantly received subsequent chemotherapy (8 out of 21, 38.1%), whereas patients in the chemotherapy group predominantly received subsequent immunotherapy (33 out of 102, 32.35%).

Information on subsequent second‐line, third‐line and fourth‐line anticancer therapy is reported in Table 5.

**TABLE 5:** Subsequent therapy.

Subsequent 2 L therapy, *N* (%)	Immunotherapy with or without chemotherapy *N* = 9 (42.8%)	Chemotherapy *N* = 59 (57.8%)
Any subsequent anti‐PD‐(L)1 therapy	1 (11.11%)	33 (55.9%)
Combination immunotherapy with chemotherapy	1 (11.11%)	1 (1.69%)
Single Immunotherapy	0 (0%)	32 (54.24%)
Any subsequent chemotherapy	8 (88.88%)	26 (44.06%)
Platinum‐doublet	1 (11.11%)	4 (6.78%)
Single agent	0 (0%)	22 (37.29%)
Other agent	7 (77.78%)	0 (0%)

Subsequent 3 L therapy, *N* (%)	Immunotherapy with or without chemotherapy *N* = 3 (14.2%)	Chemotherapy *N* = 22 (21.5)
Any subsequent anti‐PD‐(L)1 therapy	0 (0%)	1 (4.55%)
Single Immunotherapy	0 (0%)	1 (4.55%)
Any subsequent chemotherapy	3 (100%)	21 (95.45%)
Platinum‐doublet	1 (33.33%)	5 (22.73%)
Single agent	2 (66.67%)	16 (72.73%)

Subsequent 4 L therapy, *N* (%)	Immunotherapy with or without chemotherapy *N* = 1 (4.7%)	Chemotherapy *N* = 3 (2.9%)
Any subsequent anti‐PD‐(L)1 therapy	1 (100%)	0 (0%)
Single Immunotherapy	1 (100%)	0 (0%)
Any subsequent chemotherapy	0 (0%)	3 (100%)
Single agent	0 (0%)	3 (100%)

### 3.3 Safety

Adverse events were reported across all patients in both cohorts. Commonly observed AEs included anemia in 81% of immunotherapy‐based group and 92% in chemotherapy group, neutropenia (19% and 52%, respectively), thrombocytopenia (19% and 29%, respectively), dyspnea (43% and 48%, respectively), peripheral neuropathy (14% and 17%, respectively), and hepatitis (14% and 30%, respectively). Notably, immune‐related AEs were more frequently observed in the immunotherapy‐based group compared to the chemotherapy group.

Specifically, immune‐mediated pneumonitis occurred in one patient in the immunotherapy‐based group (5%), graded as 1, while none were reported in the chemotherapy group. Adverse events of grade 3 or higher included anemia (24% in the immunotherapy‐based group vs. 22% in the chemotherapy group), neutropenia (5% vs. 8%, respectively), thrombocytopenia (0% vs. 7%, respectively), and hepatitis (10% vs. 0%, respectively). Fortunately, no fatal AEs were documented in either group. The details of adverse events are shown in Table 6.

**TABLE 6:** Adverse events.

Adverse events	Immunotherapy with or without chemotherapy (*N* = 21)			Chemotherapy (*N* = 102)		
	Any grade	Grade 1–2	Grade 3–5	Any grade	Grade 1–2	Grade 3–5
Hematologic						
Anemia	17 (80.95%)	12 (57.14%)	5 (23.81%)	94 (92.15%)	72 (70.59%)	22 (21.57%)
Neutropenia	4 (19.04%)	3 (14.29%)	1 (4.76%)	53 (51.96%)	45 (44.12%)	8 (7.84%)
Thrombocytopenia	4 (19.05%)	4 (19.05%)	0	30 (29.41%)	23 (22.55%)	7 (6.86%)
Febrile neutropenia	0	0	0	2 (1.96%)	1 (0.98%)	1 (0.98%)
Non‐hematologic						
Nausea	0	0	0	5 (4.9%)	5 (4.9%)	0
Vomiting	0	0	0	3 (2.94%)	3 (2.94%)	0
Decrease appetite	2 (9.52%)	2 (9.52%)	0	37 (36.27%)	37 (36.27%)	0
Dyspnea	9 (42.86%)	9 (42.86%)	0	49 (48%)	47 (46.08%)	2 (1.96%)
Peripheral edema	2 (9.52%)	2 (9.52%)	0	2 (1.96%)	2 (1.96%)	0
Peripheral neuropathy	3 (14.29%)	3 (14.29%)	0	17 (16.67%)	17 (16.67%)	0
Diarrhea	0	0	0	0	0	0
Fever	0	0	0	1 (0.98%)	1 (0.98%)	0
Hepatitis	3 (14.29%)	1 (4.76%)	2 (9.52%)	31 (30.39%)	31 (30.39%)	0
irAEs						
Hypothyroidism	7 (33.33%)	7 (33.33%)	0	5 (4.9%)	5 (4.9%)	0
Hyperthyroidism	0	0	0	0	0	0
Pneumonitis	1 (4.76%)	1 (4.76%)	0	0	0	0
Myocarditis	0	0	0	0	0	0
Hepatitis	3 (14.28%)	1 (4.76%)	2 (9.52%)	0	0	0
Encephalitis	0	0	0	0	0	0
Thyroiditis	2 (9.52%)	2 (9.52%)	0	0	0	0

## 4 DISCUSSION

In this retrospective observational cohort study conducted at a tertiary care hospital in an upper‐middle‐income country. It was found that patients receiving immunotherapy with or without chemotherapy experienced significantly improved PFS compared to those treated with chemotherapy alone as first‐line therapy for advanced stage NSCLC. However, no significant difference was observed in OS between the two groups after adjusted in multivariate analysis. OS was significant different in patients who was received immunotherapy in their lifetime either in univariate or multivariate analysis.

In the primary endpoint, immunotherapy‐based group showed a median PFS of 11.9 months compared to 5.9 months in the chemotherapy group (adjusted HR 0.45; 95% CI, 0.25–0.81; *p* = 0.001). For the key secondary endpoint, immunotherapy‐based regimen demonstrated a median OS of 22.6 months versus 11.2 months in the chemotherapy group (adjusted HR 0.64; 95% CI, 0.29–1.38; *p* = 0.261). Despite the promising OS with immunotherapy‐based regimen, there was an initial steep decline in the survival curve, mainly due to patient deaths in the early months, especially in those receiving single immunotherapy without chemotherapy. These findings are consistent with previous studies^8^, ^13^ on single immunotherapy and underscore the importance of carefully selecting patients for appropriate treatment modalities.

Histologic subtypes like large cell carcinoma and poorly differentiated carcinoma exhibited shorter OS, in line with their recognized poor prognostic factors.^32^, ^33^, ^34^ Patients with metastases to the lungs, liver, brain, and adrenal glands typically had shorter OS compared to those without such metastases.^35^, ^36^, ^37^ This could be due to a higher organ metastasis, although it is difficult in defining tumor burden accurately.

In the multivariate analysis of OS, after adjusting for covariates between the immunotherapy‐based and chemotherapy groups, revealed no significant different in OS (HR for death 0.64; 95% CI, 0.29–1.38; *p* = 0.261). Consequently, further analysis of OS by metastatic site was not pursued due to multicollinearity. If the definition of high tumor burden is well described in the future, we think that tumor burden can affect patient survival and should be considered in the adjusted model.

In BMI analysis, patients with normal 18.5–24.9 kg/m^2^or mildly elevated BMI 25–29.9 kg/m^2^showed improved OS compared to those with low BMI, likely due to better nutritional status and ECOG‐PS, a recognized prognostic factor in cancer.^38^, ^39^

Additionally, since 65% of patients received second‐line single immunotherapy, it may affected have impacted OS because second‐line immunotherapy also provides survival benefit compared to chemotherapy.^40^, ^41^, ^42^

The consistent and favorable outcomes with immunotherapy, irrespective of PD‐L1 status, highlight its effectiveness in treating non‐driver advanced NSCLC.^5^ The safety profile of immunotherapy aligned with prior research,^6^, ^7^, ^8^, ^9^, ^10^, ^11^, ^12^, ^13^, ^14^, ^15^, ^43^ primarily exhibiting low‐grade adverse events, allowing patients to continue treatment.

Comparison with pivotal studies such as KEYNOTE‐024,^7^ KEYNOTE‐042,^13^ and IM power‐110,^9^ which utilize single immunotherapy as first‐line treatment, same as eight patients in our study who received single immunotherapy, they showed similar improvements in both PFS (7.3 months) and OS (23.4 months) as observed in these pivotal studies. For example, in KEYNOTE‐042, pembrolizumab monotherapy demonstrated benefits in patients with PD‐L1 Positive (TPS greater than 1%), with median PFS of 6.5 months, median OS of 20 months, and ORR of 40%. However, subgroup analysis revealed no clear survival benefit in patients with PD‐L1 TPS 1%–49%. Similarly, IMpower‐110 showed the advantage of first‐line atezolizumab monotherapy in PD‐L1 TC3/IC3 groups, with a median PFS of 8 months, median OS of 20 months, and ORR of 40%, while no benefit was observed in other PD‐L1 subgroups.

Exploring pivotal studies of first‐line combination immunotherapy with chemotherapy, like KEYNOTE‐189,^15^ KEYNOTE‐407,^43^ IMpower‐150,^8^ and IM power‐130,^11^ revealed comparable survival benefits to patients receiving combination immunotherapy with chemotherapy in our study (median PFS 11.9 months and median OS not reach). For example, KEYNOTE‐189 and KEYNOTE‐407, which encompassed patients with PD‐L1 all comers but different histologic subtypes (adenocarcinoma and squamous cell carcinoma, respectively), showed improved median PFS to 9 and 8 months, median OS to 22 and 17 months, and ORR to 48.3% and 62%, respectively, with first‐line combination pembrolizumab with chemotherapy. Similarly, IMpower‐130, including nonsquamous PD‐L1 all comers using first‐line combination atezolizumab with chemotherapy, exhibited median PFS of 7 months, median OS of 18 months, and ORR of 50%.

Across toxicity profiles, either immunotherapy with or without chemotherapy patients in our study yield similar toxicity results as previously reported, real‐world data and pivotal studies.

In many real‐world studies,^20^, ^21^, ^22^, ^23^, ^24^ outcomes comparable to RCTs have been observed, despite including patients with poor prognostic factors such as high tumor burden, unstable or untreated CNS metastasis, and ECOS‐PS greater than 2^19^ who are typically excluded from RCTs. In immunotherapy‐based group, 15% of patients had ECOG‐PS greater than 2, and 24% had CNS metastasis at baseline.

These observations emphasize the benefits of first‐line immunotherapy, whether alone or in combined with chemotherapy, as shown in our study, with survival outcomes comparable to pivotal trials. Subgroup analysis of OS underscores prolonged survival with immunotherapy across treatment lines.

Our study's strength lies in being the first real‐world dataset from Thailand, examines patients receiving first‐line immunotherapy with or without chemotherapy compared to chemotherapy alone. It demonstrates comparable survival benefits and safety to pivotal studies, providing crucial insights into information on the survival benefits for patients receiving immunotherapy across any treatment lines.

However, there are limitations. First, the small sample size in the immunotherapy‐based group, potentially from single‐center data, may impact survival outcomes and lead to poor accuracy. Second, accurately defining tumor burden presents challenges, hindering the ability to fully understand the potential impact of metastatic site and tumor burden on survival outcomes.

In conclusion, our study present real‐world retrospective observational cohort highlights the clinical benefits and safety of administering first‐line immunotherapy with or without chemotherapy to patients with advanced NSCLC that define in PFS benefit but not OS after adjusted multivariate analysis. These findings offer valuable insights into the decision to use immunotherapy as a treatment for NSCLC, for treatment decisions however some patients may have not opportunity to receive immunotherapy due to poor tolerability, poor performance status, and disease progression and provide valuable data for healthcare systems in regions with limited resources, like Thailand which was upper middle income country compared to many developed countries.

## AUTHOR CONTRIBUTIONS

**Danainut Naratornsirakul:** Conceptualization (lead); data curation (equal); formal analysis (equal); investigation (equal); methodology (lead); validation (equal); writing – original draft (equal); writing – review and editing (equal). **Busyamas Chewaskulyong:** Conceptualization (equal); data curation (equal); formal analysis (equal); investigation (equal); methodology (equal); project administration (lead); resources (equal); supervision (lead); validation (equal); visualization (lead); writing – original draft (equal); writing – review and editing (equal). **Sarawut Kongkarnka:** Resources (equal). **Songporn Oranratnachai:** Data curation (equal); formal analysis (equal); writing – original draft (equal); writing – review and editing (equal).

## FUNDING INFORMATION

This research had no funding.

## CONFLICT OF INTEREST STATEMENT

BC Disclosure: The speaker and researchers have affiliations with MSD, the speaker with BMS, and both the research and speaker with Roche. DN, SK,SO declare no conflicts of interest.

## INSTITUTIONAL REVIEW BOARD STATEMENT

The study was conducted in accordance with the Declaration of Helsinki and approved by Research Ethics Committee Panel 5, Faculty of Medicine, Chiang Mai University (MED‐2566‐0338, approval date 23 August 2023).

## INFORMED CONSENT STATEMENT

Informed consent was not obtained due to the study's retrospective nature, and patient names weren't disclosed. The need for consent was waived as per IRB approval for using de‐identified medical records.

## Data Availability

The data that support the findings of this study are available from the corresponding author upon reasonable request. This study utilized data available from the Maharaj Nakorn Chiang Mai Hospital database spanning from January 2013 to December 2023.
